# Antimalarial Potential of* Carica papaya* and* Vernonia amygdalina* in Mice Infected with* Plasmodium berghei*

**DOI:** 10.1155/2016/8738972

**Published:** 2016-11-29

**Authors:** Oche Okpe, Nathan Habila, Joseph Ikwebe, Vincent A. Upev, Stanley I. R. Okoduwa, Omiagocho T. Isaac

**Affiliations:** ^1^Department of Biochemistry, University of Agriculture, Makurdi, Nigeria; ^2^Department of Biochemistry, Ahmadu Bello University, Zaria, Nigeria; ^3^Directorate of Research and Development, Nigerian Institute of Leather and Science Technology, Zaria, Nigeria

## Abstract

The study determined if administration of* Vernonia amygdalina* and* Carica papaya* plants provides synergistic effects in ameliorating plasmodium infection in mice. Thirty mice (17.88–25.3 g) were divided into 6 groups of 5 mice each. Group 1 was normal control, while groups 2–6 were intraperitoneally inoculated 2.5 × 10^7^ *Plasmodium berghei *parasitized red blood cell, followed by daily administration of 350 mg/kg aqueous leaf extracts after establishment of infection. Group 2 was disease control, while group 6 was treated with standard drug for four consecutive days. The results showed significant (*P* < 0.05) reduction in percentage of parasite load between the infected treatment groups and disease control group at day 3 after infection, which remained consistent until the end of the experiment. All infected treated groups showed significant (*P* < 0.05) increases in RBC and PCV recovery compared to the disease control, with the exception of WBC. There was insignificant (*P* > 0.05) change in mean body weight of all treated groups except in disease control group. Histological studies of the infected mice indicate recovery of hepatic cells from congested black pigmentation. The reduction in parasite load and recovery of hepatic cell damage/hematological parameters were induced by these plant extracts. This highlighted the important usage of the plant in traditional remedy of malaria infection.

## 1. Introduction

Malaria remains one of the deadliest infectious diseases, caused by protozoan parasite of the genus* Plasmodium *[1]. Available therapeutic agents are already limited in their efficacy, while drug resistance threatens the ability to prevent and treat the infection [1]. Malaria is the leading cause of illness and death in sub-Saharan Africa with an annual mortality of approximately one million children under five [2]. In Nigeria and the rest of the world, malaria infection continues to pose a major health challenge. In view of resistance of the parasite to antimalarial drug therapy, which leads to drug failure, new drugs or drug combinations are urgently required for the treatment of malaria infections from traditional medicinal plants [3]. Traditional medicinal plants play an important role in the medical system in Nigeria; however, plant materials remain an important resource to combat serious diseases in the world. Currently used plants have always been considered to be a possible alternative and rich source of new drugs, and some of the antimalarial drugs in use today such as quinine and artemisinin were either obtained directly from plants or developed using chemical structures of plant-derived compounds as templates [4].

The uses of synthetic antimalarial drugs are among the main ways to treat malaria and are effective in controlling parasite load. However, the practical application of the majority of these therapeutic agents remains restricted owing to their limited action, pharmacokinetic properties, secondary failure rate, and accompanying side effects [5]. With the increasing incidence of the disease in urban/rural population throughout the world, there is clear need for development of indigenous, inexpensive botanical sources for antimalarial crude or purified drugs [5].

*Vernonia amygdalina *Del belongs to the family Asteraceae; its common name is bitter leaf. It is a perennial shrub of 2–5 m in height that grows throughout tropical Africa [6]. The leaf and stem are acclaimed to be one of the most used medicinal plants in traditional practice [7].* Carica papaya Linn* belongs to the Caricaceae family and is commonly known as pawpaw [8]. The leaf of* Carica papaya* in combination with* Vernonia amygdalina* is used traditionally to treat infection [7, 9]. In traditional medicine, herbal practitioners use* V. amygdalina* and* C. papaya *in Nigeria as anthelmintic, antimalaria, digestive tonic, appetizer, antihemolytic, antifungal, and antijaundice agents. The leaf infusion is used in treatment of urinary disorders and gonorrhea. The fresh leaves are used in dressing of wounds or as antidysentery agent and against chronic diarrhea or as sedative and as cure for pile and wounds of the urinary tracts and several other ailments [9]. The plant extracts have been scientifically proven to function as antibacterial [7], antifungal [7, 10], anticancer/tumor [11, 12], and antiplasmodial agents [13, 14].

While the physiological role of some plant active principle(s) has been intensively studied and their functions in health and disease conditions have been recognized, it is still a focus of pharmaceutical research in the development of agent that can effectively treat malaria and many other diseases, hence the need to study the efficacy of* V. amygdalina* and* C. papaya* in living systems in order to foster information regarding its medical applications in malarial infection.

## 2. Materials and Methods

### 2.1. Plant Samples Collection and Identification

Fresh leaves of* Vernonia amygdalina* and* Carica papaya* were collected in November 2015, from Federal Low Cost Housing Estate, Makurdi, Nigeria. The plants were identified and authenticated by a taxonomist in the Department of Biological Sciences, University of Agriculture, Makurdi, Nigeria.

### 2.2. Experimental Animals

Adult mice (17.88–25.3 g) of both sexes were obtained from the Laboratory Animal House, College of Health Sciences, Benue State University, Makurdi, Nigeria. The animals were acclimatized for 2 weeks under standard environmental conditions. The temperature and humidity were maintained at 25°C and 50%, respectively. Dark and light cycles were maintained at 12 hrs each. They had access to standard commercial rat pellets (UAC Grand Cereal Ltd., Jos, Nigeria) and water* ad libitum*. The animals were used in accordance with the guideline and recommendation of the ethics committee on the use of animals for research of the University of Agriculture, Makurdi, Nigeria.

### 2.3. Equipment/Reagents

The following equipment and reagents were used: Olympus microscope, hemocytometer with aspirating pipette, capillary tube, microhematocrit centrifuge, PCV reader, cover slip and slides, and water bath. Turk's fluid for WBC, Dacie's fluid for RBC, and Giemsa stain were purchased from Sigma-Andrich Chemical Company (St. Louis, USA). All other chemicals and reagents used for this study were of analytical grade.

### 2.4. Preparation of Plant Sample

The collected samples were rinsed in clean water and shade dried at room temperature for two weeks. The dry plants sample was ground into powder using pestle and mortar. The powder obtained was then used to prepare the extracts.

### 2.5. Aqueous Extraction

Exactly 100 g of the dried powder was weighed out and soaked in the solvent (water) with the solute-solvent proportion of 1 : 10. The extraction was by cold maceration at room temperature with intermittent shaking at 3-hour intervals for 48 hours. Whatman filter paper of 8 *μ*m was used to filter the extract. The filtrate obtained was concentrated in temperature regulated water bath at 45°C.

### 2.6. Lethality (LD_50_) Test

The mean lethal dose (LD_50_) of the aqueous extracts was determined in mice (weighing 20–30 g) using the arithmetic-geometric-harmonic (AGH) methods of rough estimation as modified by Saganuwan [15].

### 2.7. Malaria Parasite

The malaria parasite (Chloroquine sensitive* Plasmodium berghei* (Nk65)) was obtained from the Faculty of Veterinary Medicine, Ahmadu Bello University (ABU), Zaria.

### 2.8. Parasite Inoculation

*Plasmodium berghei* parasitized erythrocytes were obtained from the tail of the donor mice and were diluted with 0.9% normal saline. Mice were inoculated intraperitoneally with 0.5 mL blood suspension containing 2.5 × 10^7^ parasitized erythrocytes on day 0 and were monitored for manifestation of parasitemia for 4 days without treatment. The mice were randomly divided into 6 groups of five (5) mice per group and treated for 4 consecutive days with daily doses of the extracts (350 mg/kg b.w) and standard antimalarial drug (halofantrine, 25 mg/kg b.w) by oral route.

### 2.9. Animal Grouping and Treatment

Group 1: negative control, not infected with* P. berghei *and not treated
Group 2: positive control, infected with* P. berghei* but not treated
Group 3: infected with* P. berghei *and treated with 350 mg/kg* C. papaya* extract
Group 4: infected with* P. berghei *and treated with 350 mg/kg* V. amygdalina *extract
Group 5: infected with* P. berghei *and treated with 350 mg/kg combined extracts of* C. papaya* and* V. amygdalina *in ratio 1 : 1
Group 6: infected with* P. berghei* and treated with antimalarial drug (halofantrine, 25 mg/kg)

### 2.10. Hematological Analysis

The percentages of parasitized erythrocytes levels were determined as described by Brown [16], using a microscopic examination of thin blood smears made on microscopic slide. The packed cell volume was assayed according to the method described by Coles [17]. The RBC and WBC count was estimated according to the protocol of Brown [16], using the Neubauer haemocytometer.

### 2.11. Histological Analysis

At the end of the experiment, all the mice were anaesthetized using chloroform and bled by cardiac puncture. The hepatic tissues were dissected out of all the mice and washed on ice cold saline immediately. A portion of the tissue was fixed in 10% formalin fixative solution for histological studies as described by Strate et al. [18].

### 2.12. Statistical Analysis

The analysis was carried out in triplicate for all determinations and the results were expressed as mean ± SEM. The SPSS program (version 20.0 SPSS Inc., Chicago, IL, USA) was used for the analysis of variance, followed by the new Duncan's multiple range tests for multiple comparisons of the means [19]. *P* < 0.05 between mean values was considered statistically significant.

## 3. Results

**Figure 1:**
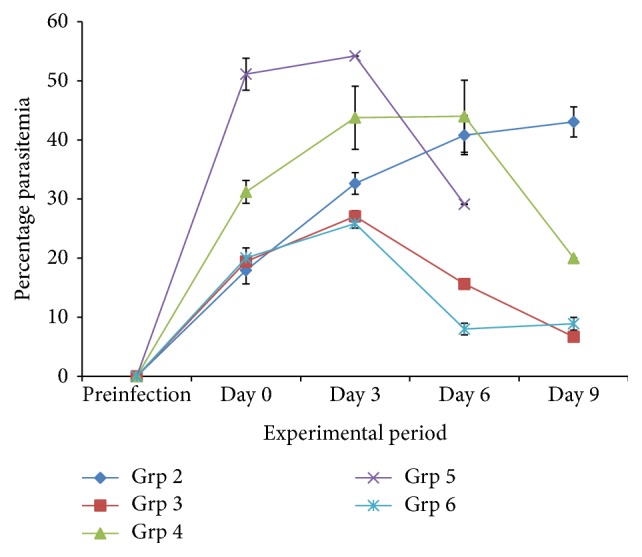
The percentage of parasitized erythrocytes in mice infected with* P. berghei *and treated with aqueous leaf extracts of* V. amygdalina* and* C. papaya,* and halofantrine.

The results of this study show that aqueous leaf extracts of* V. amygdalina* and* C. papaya* displayed antimalarial activity in an infected mouse when compared to the infected, untreated control.  Figure 1 reveals the percentage of parasitized erythrocytes of mice infected with* P. berghei. *The groups infected with* P. berghei* developed parasitemia after infection. The percentage of parasitized erythrocytes in the infected untreated group (Group 2) was significantly (*P* < 0.05) increased on day 9 compared to postinfection day (within group) and other groups (between groups), respectively. The significant (*P* < 0.05) reduction in parasite load was more pronounced in groups 3 and 6, respectively.

Table 1 shows the mean RBC, PCV, and WBC count of mice infected with* P. berghei* and subsequently treated with aqueous extracts of* C. papaya, V. amygdalina, *combined* C. P. + V. A.,* and halofantrine. There was significant (*P* < 0.05) decrease in mean RBC count and PCV in all the experimental groups after infection compared to normal control group. However, as treatment commenced, there was an observed significant (*P* < 0.05) increase in mean RBC count and PCV within groups in all the treated groups (except RBC in group 4) compared to the infected, untreated control group. The mean white blood cells count of all the groups infected with* P. berghei* was significantly (*P* < 0.05) increased after infection compared to the normal control. Increase in WBC was significantly reduced upon administration of the different regimen of extracts to infected groups.

**Figure 2:**
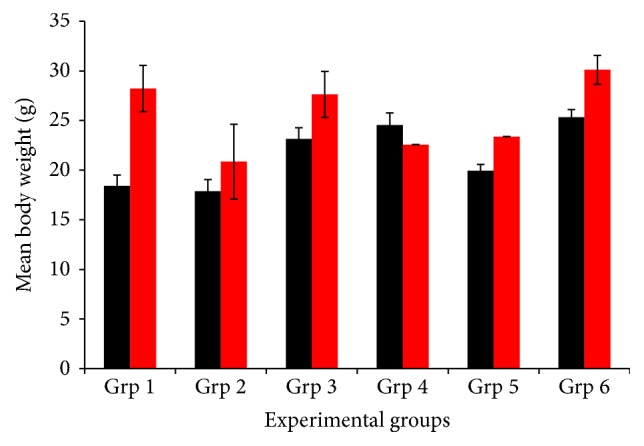
The mean body weight of mice infected with* P. berghei *and treated with aqueous leaf extracts of* V. amygdalina*,* C. papaya,* and halofantrine. Black colour indicates preinfection; red colour indicates day 9, except in Grp 5 (indicating day 6).

The body weight of all the experimental groups was presented in Figure 2. There was significant (*P* < 0.05) increase in the body weight gain throughout the postinfection period in groups 1, 3, and 6 compared to the preinfection day. However, no significant increase was observed in groups 2, 4, and 5 when compared to the preinfection day.

**Figure 3:**
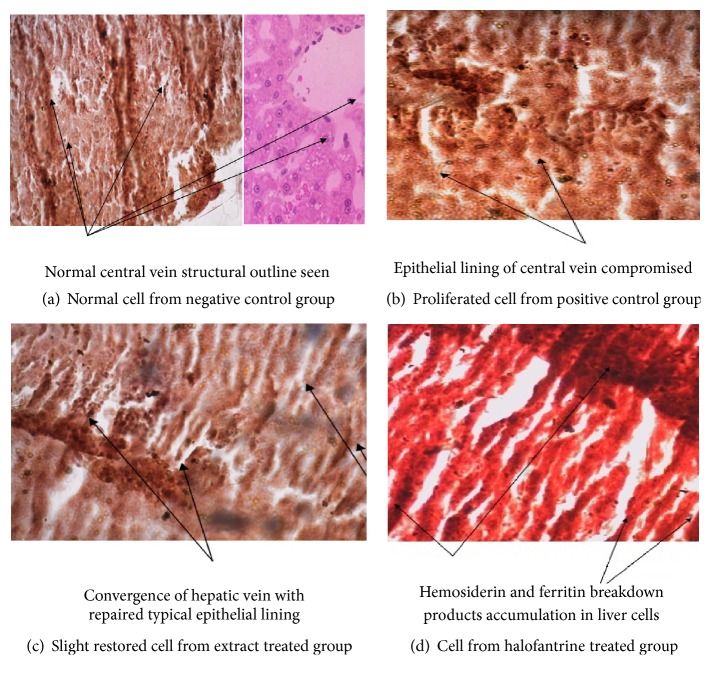
Representative central vein of the hepatic cells of the parasitic mice treated with leaf extracts of* V. amygdalina, C. papaya,* and halofantrine.

Figure 3 shows the histological section of the hepatocyte of mice infected with* P. berghei *and treated with aqueous extracts of* C. papaya, V. amygdalina, *combined* C. P. + V. A.,* and halofantrine.  Figure 3(a) is the normal control mice with normal architecture of hepatic cells and reference standard.  Figure 3(b) is the malarial control mice with complete depletion of hepatic cells by* P. berghei*. Here, the figure revealed prominent sinusoids and sinusoidal spaces, along with areas of necrosis.  Figure 3(c) is the representative hepatocyte from infected mice treated with 350 mg/kg b.w of the extracts. In this figure, there was a slight restoration of diffused proliferated hepatic cells.  Figure 3(d) is the infected mice treated with 25 mg/kg b.w halofantrine; this shows slight restoration of diffused proliferated hepatic cells.

## 4. Discussion

The existence of experimental animal models of a disease aids not only in the understanding of the pathophysiology of such disease, but also in the development of drug candidate [20].

Therefore, screening for antimalarial activity of plant crude extracts is the first step in isolation of new molecules with potent activity [21, 22]. The results clearly indicate that the individual administration of aqueous leaf extract of* C. papaya*,* V. amygdalina, *and the combination of both plants significantly (*P* < 0.05) decreased parasite load in mice and enhanced their survival. There are many bioactive constituents present in both extracts and hence, at present, it is not certain, which is/are responsible for the observed effects. However, some reports have shown that flavonoids, tannins, saponins, and other phytoconstituents may play some roles in the inhibition of malaria parasites in infected animals [23, 24]. The observed parasitemia in the experimental mice when treated separately with single doses and with a combined dose of* C. papaya* and* V. amygdalina* synergistically decreases along with halofantrine on day 9 after establishment of infection, compared with the disease control which is characterized by excessive parasite growth, exaggerated immune reactions, or a combination of both causing severe pathology and death, which is detrimental to both parasite and host. Thus, the findings of Saganuwan et al. [25] that many Nigerian plants can be used for the treatment of malaria and that infection with* P. berghei* is almost fatal with death occurring within 1–3 weeks are corroborated[25].

The decrease in PCV and RBC and increase in WBC of all the infected groups after infections are in conformity with previous reports, documenting reduced serum PCV, RBC, and an increased WBC levels in malarial subjects [5]. In this study, administration of aqueous leaf extracts of* C. papaya, V. amygdalina,* and the combined extracts significantly reduced WBC and triggered an increase in PCV as a result of increased production of RBC, thus, suppressing hemolytic damage to RBC.

The increase in body weight of the infected groups of mice and the noninfected group disagrees with the report of Saganuwan and Onyeyili [5], indicating that* P. berghei* decreases body weight. The increase in body weight observed in this study may be due to constant feeding of the animals during the experimental period.

Histological examination of the liver cells in the treated groups showed the inability of the extract to clear the entire parasite inclusion from circulation and repair the hepatocyte, which agrees with the work of Stenad et al. [26], who stated that hepatic inclusions are defined as intracellular aggregates of stainable substances which represent established hallmarks of their respective human disorder. Signs of regeneration/repairs of tissues using medicinal plants have been reported in other works [27, 28], which are consistent with the present study. Thus, tissue repairs or therapy may offer therapeutic benefit in disease conditions.

## 5. Conclusion

In conclusion, the finding revealed that the reduction in parasite load and recovery of hepatic cell damage/hematological parameters were induced by the plant extracts and thus gave credence to the usage of the plant traditionally for the treatment of malaria.

## Figures and Tables

**Table 1:** The red blood cell and white blood cell count of mice infected with *P. berghei *and treated with aqueous leaf extracts of *V. amygdalina*, *C. papaya,* and halofantrine.

Parameters	Groups	Preinfection	Day 0	Day 3	Day 6	Day 9
RBC (×10^12^/l)	GRP I	13.60 ± 0.47^b^	13.08 ± 0.53^b^	11.70 ± 0.74^ab^	10.88 ± 0.27^a^	11.48 ± 0.62^a^
	GRP II	12.10 ± 0.70^c^	6.50 ± 1.92^b^	1.23 ± 0.21^a^	1.40 ± 0.51^a^	2.15 ± 0.33^a^
	GRP III	9.84 ± 0.49^c^	5.44 ± 0.24^b^	2.90 ± 0.05^a^	4.23 ± 0.11^a^	5.23 ± 0.18^b^
	GRP IV	10.26 ± 0.47^c^	5.42 ± 0.72^b^	2.90 ± 0.72^a^	2.37 ± 0.29^a^	5.57 ± 0.23^b^
	GRP V	12.80 ± 1.37^c^	4.42 ± 0.19^b^	0.93 ± 0.23^a^	2.30 ± 0.31^a^	
	GRP VI	11.56 ± 0.21^e^	2.70 ± 0.22^b^	1.63 ± 0.27^a^	5.45 ± 0.17^c^	6.45 ± 0.22^d^

PCV (%)	GRP I	50.40 ± 1.46^b^	44.20 ± 2.33^ab^	44.20 ± 4.80^ab^	46.20 ± 1.95^ab^	38.60 ± 2.15^a^
	GRP II	52.00 ± 1.87^d^	43.20 ± 0.73^c^	30.33 ± 4.37^b^	33.50 ± 1.50^b^	17.50 ± 2.50^a^
	GRP III	48.60 ± 1.40^c^	43.20 ± 0.73^c^	20.75 ± 1.85^a^	41.50 ± 2.53^bc^	32.75 ± 5.34^b^
	GRP IV	43.20 ± 2.52^b^	44.60 ± 0.81^b^	29.50 ± 2.06^a^	26.33 ± 5.78^a^	30.00 ± 0.00^a^
	GRP V	47.20 ± 1.71^d^	39.20 ± 1.24^c^	23.00 ± 0.00^b^	18.00 ± 0.00^a^	
	GRP VI	49.80 ± 0.48^d^	34.80 ± 1.85^b^	27.50 ± 2.90^a^	46.50 ± 1.50^cd^	45.50 ± 0.50^c^

WBC (×10^9^/l)	GRP I	4.68 ± 0.59^ab^	4.32 ± 0.56^a^	4.56 ± 0.55^ab^	7.04 ± 0.32^bc^	6.04 ± 0.63^c^
	GRP II	4.20 ± 1.86^a^	6.75 ± 1.24^a^	17.93 ± 4.45^a^	13.00 ± 0.25^ab^	13.20 ± 0.38^b^
	GRP III	6.08 ± 0.83^a^	6.30 ± 0.59^ab^	8.13 ± 1.75^ab^	6.73 ± 1.17^b^	8.93 ± 0.38^b^
	GRP IV	6.36 ± 1.32^ab^	5.92 ± 1.16^a^	11.27 ± 1.89^c^	10.33 ± 5.34^bc^	5.80 ± 0.00^ab^
	GRP V	8.00 ± 1.76^a^	13.90 ± 0.65^b^	15.2 ± 0.00^b^	7.60 ± 0.00^a^	
	GRP VI	4.68 ± 1.42^a^	15.36 ± 1.42^c^	12.10 ± 1.89^b^	4.60 ± 0.51^ab^	7.80 ± 0.13^ab^

Values are expressed as mean ± SEM of five replicate determinations. Values with different superscript along the rows are significantly different at *P* < 0.05.
